# High-Grade Transformation (Dedifferentiation) of Acinic Cell Carcinoma of the Parotid Gland: Report of an Unusual Variant

**DOI:** 10.1155/2017/7296467

**Published:** 2017-05-14

**Authors:** Sarah S. Al-Otaibi, Faiza Alotaibi, Yaseer Al Zaher, Nabil Al Zaher, M. A. Dababo

**Affiliations:** ^1^Department of Otolaryngology, Head & Neck Surgery, King Faisal Specialist Hospital & Research Centre, MBC 47, P.O. Box 3354, Riyadh 11211, Saudi Arabia; ^2^Department of Pathology, King Faisal Specialist Hospital & Research Centre, P.O. Box 3354, Riyadh 11211, Saudi Arabia

## Abstract

Acinic cell carcinoma with high-grade transformation of the salivary gland is an unusual variant with less than fifty cases being reported in the literature. It is characterized by a low- and high-grade component juxtaposed with one another and tends to take on a more aggressive clinical course than its low-grade counterpart, suggesting a poor clinical outcome. We, hereby, report a case of acinic cell carcinoma in a 48-year-old woman with a 6-month history of a right parotid facial swelling rapidly increasing in size. The tumor was initially resected; however, residual focal tissue subsequently revealed areas typical of low-grade acinic cell carcinoma as well as high-grade transformation/dedifferentiation via histopathology.

## 1. Introduction

Acinic cell carcinoma (ACC) of salivary gland represents 2.5%–5% of all parotid gland neoplasms [1]. It was identified as a separate entity more than a century ago, and its malignant potential was first ascribed by Buxton et al. [2]. ACC is a low-grade malignant tumor commonly affecting the parotid gland followed by the minor salivary glands and is typically seen among young females [3].

An exceedingly peculiar variant of this tumor has been described as acinic cell carcinoma with high-grade transformation/dedifferentiation which is characterized by the coexistence of both low-grade acinic cell carcinoma and a high-grade dedifferentiated component, as well as by an accelerated clinical course. The high-grade transformation/dedifferentiation delineates a more aggressive behavior and is associated with a poorer prognosis than its traditional counterpart [4].

The first salivary gland case with high-grade transformation/dedifferentiation was reported in 1988 by Stanley et al. [4] describing a dedifferentiated acinic cell carcinoma of the parotid gland. Since the original report by Stanley et al. [4] in 1988, only a few cases of dedifferentiated acinic cell carcinoma have been described or briefly mentioned in the English literature [3, 5–8]. Other salivary gland carcinomas with high-grade transformation have been documented as well, including adenoid cystic carcinoma [9], epithelial-myoepithelial carcinoma [10], and mucoepidermoid carcinoma [11].

In this article, we report a case of a parotid gland acinic cell carcinoma exhibiting features of high-grade transformation and describe its clinicopathological aspects while shedding some light on its diagnosis and management.

## 2. Case Presentation

A 48-year-old woman presented with a 6-month history of a right parotid swelling that rapidly and progressively increased in size. This was associated with occasional local pain and facial asymmetry. She was diagnosed, at another institution, with salivary duct carcinoma with multifocal perineural and intraneural invasion and underwent partial parotidectomy. The patient was subsequently referred to our hospital for further management due to questionable positive surgical margins. Her evaluation demonstrated a recent surgical scar indicating a previous parotidectomy incision along with grade V facial palsy.

**Figure 1:**
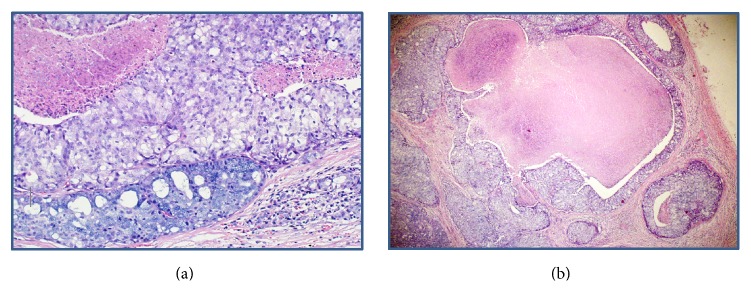
(a) Hematoxylin and eosin stain illustrating a low-grade acinic cell carcinoma at the bottom juxtaposed to a high-grade carcinoma, simulating a salivary duct carcinoma with central necrosis. (b) An area of high-grade transformation demonstrating an infiltrating tumor with extensive central necrosis.

**Figure 2:**
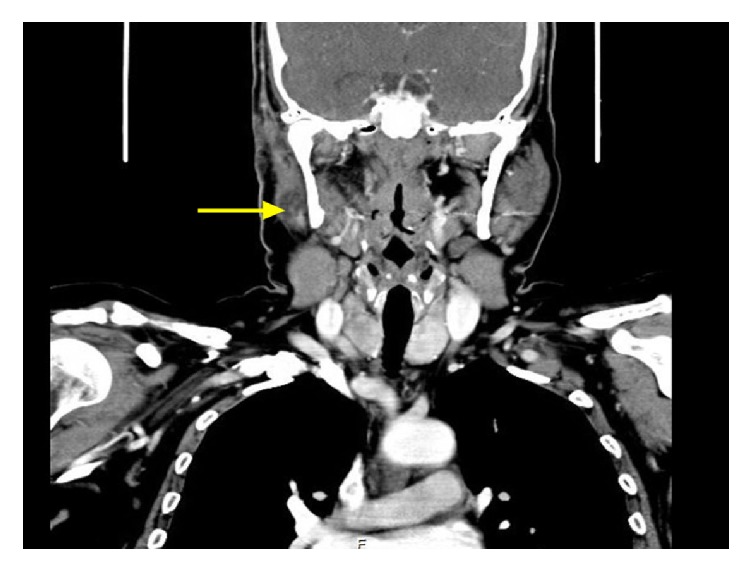
Coronal CT image demonstrates the residual parotid tumor (arrow), before the definitive surgery.

Pathology slides were reviewed and revealed acinic cell carcinoma with areas of high-grade transformation (Figures 1(a) and 1(b)). Multifocal perineural and intraneural invasion were also noted; however, the margins were positive. Computed Tomography (CT) scan of the head and neck showed postoperative fibrotic changes in the area of the right parotid gland (Figure 2). Pathological lymph nodes were also seen in levels 2 and 3 on the right side. CT scans of the chest, abdomen, and pelvis were unremarkable.

Positron Emission Tomography (PET) scan showed findings consistent with local residual tumor in the right parotid gland bed with metastatic jugulodigastric lymphadenopathy. There was also abnormal uptake demonstrated in the thyroid gland. The patient had an ultrasound-guided Fine Needle Aspiration (FNA) from areas with positive uptake on the PET scan; the right parotid surgical bed illustrated a low-grade epithelial neoplasm in a lymphoid background. The right jugulodigastric lymph node was reactive but negative for malignant cells and the right thyroid lobe was compatible with Hashimoto's thyroiditis. The patient underwent a right radical revision parotidectomy with right supraomohyoid neck dissection followed by a full course of radiotherapy of 6,000 cGY and 2 cycles of Cisplatin chemotherapy.

**Figure 3:**
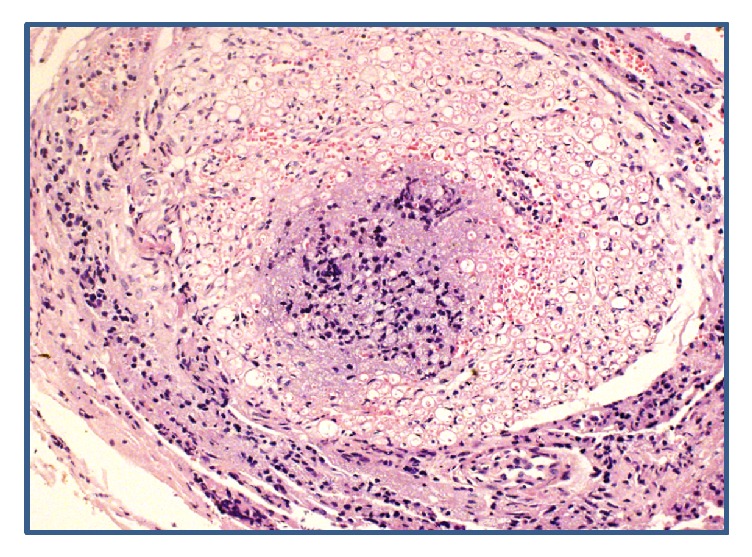
Cross section of the nerve showing endoneural invasion by the carcinoma.

The final pathology results were in agreement with the preoperative diagnosis of acinic cell carcinoma with high-grade transformation and there was clear evidence of perineural invasion (Figure 3). Metastatic carcinomas in four out of nine parotid lymph nodes and one out of thirty-eight lymph nodes were evident from the neck dissection. Fibrosis and foreign body giant cell reaction were noted, indicating a previous surgery site.

Less than a year later, the patient unfortunately developed distant metastases at multiple sites including the brain, the lungs, the liver, and the retroperitoneum in addition to multiple osseous metastatic lesions and was provided with palliative care until she passed away.

## 3. Discussion

Acinic cell carcinoma (ACC) of the salivary gland represents 2.5%–5% of all parotid gland neoplasms [1]. It was identified as a separate entity more than a century ago, and its malignant potential was first ascribed by Buxton et al. [2]. ACC is a low-grade malignant tumor commonly affecting the parotid gland followed by the minor salivary glands and is typically seen among young females [3]. The multidirectional differentiation of the neoplastic cells together with a scarcity in morphological hallmark of serous acinar cell differentiation, as evidenced in some cases, poses a real diagnostic challenge. Though the conventional ACC is a low-grade tumor, poorly differentiated and high-grade transformed variants exhibit a propensity for metastasis and an unpredictable malignant behavior [12].

The term dedifferentiation was first coined by Dahlin and Beabout [13] in their description of a peculiar clinicopathological entity of a chondrosarcoma [13], which was later established in salivary gland neoplasms [4]. The first salivary gland case was reported in 1988 by Stanley et al. [4] describing a dedifferentiated acinic cell carcinoma of the parotid gland. Other salivary gland carcinomas with high-grade transformation have been documented as well, including adenoid cystic carcinoma [9], epithelial-myoepithelial carcinoma [10], and mucoepidermoid carcinoma [11].

Dedifferentiation, or high-grade transformation (HGT), is the evolution of cells progressing from a differentiated state to a less differentiated morphological pattern. Histopathologically, a high-grade component is juxtaposed with a low-grade morphology [14]. The exact etiopathogenesis behind this phenomenon is still not clearly understood, but it has been suggested that the cells of origin either neglected to differentiate or differentiated to a high-grade morphology [14]. This incidence can occur at presentation or in a recurrent tumor [14]. Recently, Seethala et al. [15] introduced the term “high-grade transformation” which better reflects the fact that the dedifferentiated component often maintains some features of the original tumor, such as glandular differentiation [5, 15, 16].

The conventional acinic cell carcinoma is a biologically low-grade epithelial tumor that displays at least a focal serous acinar differentiation and typically exhibits a microcystic, solid, papillary-cystic, or follicular growth pattern histologically [17]. ACC is predominantly a slow-growing [18], low-grade malignancy with a female preponderance [3].

HGT ACC requires an area of HGT within an existing low-grade ACC and the rather large group of high-grade acinic cell carcinomas that do not show a preexistent low-grade part which is characterized by the presence of an increased mitotic rate (>2 mitoses/10 HPFs) [6]. The acinar architecture is lost in the high-grade component of the transformed acinic cell carcinoma. The high-grade element usually conveys solid nests of anaplastic cells with abundant cytoplasm, pleomorphic nuclei, prominent nucleoli, and brisk mitoses. It may also demonstrate a cribriform pattern, comedonecrosis, along with perineural and vascular invasion [14].

An elevated proliferative index by Ki-67 immunohistochemistry is suggestive of AiCC-HGT, but ultimately it is a morphologic diagnosis [14] and has significant prognostic value [19, 20]. P53 immunoexpression has not been found to be a reliable marker of high-grade transformation in acinic cell carcinomas [5, 21].

Skálová et al. demonstrated, in their immunohistochemical study of nine cases with HGT acinic cell carcinoma, positive expression of *β*-catenin, cytokeratin 18, and cyclin D1, whereas HER-2/*neu*, androgen receptor, and C-kit have been found to be negative in both low- and high-grade components [5]. Although acinic cell carcinomas do not typically show myoepithelial differentiation, they can rarely harbor a myoepithelial immunoprofile in the high-grade carcinomatous portions [18].

The HGT acinic cell carcinoma has been shown to be associated with aneuploidy, which is the appearance of distinct helioid inclusions ultrastructurally, whereas its low-grade counterpart conveys diploidy [12, 22].

This variant has a preponderance among females [14] with a mean age of onset around 61–66 years, exceeding that of the traditional acinic cell carcinoma by 20 years [5, 7]. While there are no definitive clinical features that present in HGT acinic cell carcinomas, Stanley et al. reported cases that presented with an enlarging, tender facial mass that may also be accompanied with difficulties in deglutition [4]. Due to its malignant behavior, it typically invades the 7th cranial nerve, manifesting with paresis, paresthesia, and paralysis of the face. This too is indicative of a dismal prognosis [23].

HGT acinic cell carcinomas have been correlated with an aggressive clinical course primarily invading the parotid gland in its entirety indicating a poorer prognosis, higher recurrence rate, and a predilection for regional lymph node and distant metastasis [1, 24]. Certain histological characteristics have been associated with frequent recurrences and metastases including multiple mitotic figures, atypical cells, and stromal hyalinization [8, 25]. It has also been reported that metastatic foci have demonstrated both low- and high-grade morphologies [14].

There are no standard guidelines on how to treat HGT ACC but because of this tumor's invasive nature and higher rate of lymph node metastasis, consideration of a total parotidectomy and lymph node dissection is reasonable, since high-grade ACC is a very aggressive malignant neoplasm that tends to be treated aggressively [26].

Adjuvant radiotherapy is recommended based on the consideration of the surgical procedure adequacy, presence of bone or perineural invasion, and clinical stage [5, 27]. The prognostic value of dedifferentiation is still to be defined, and statistically significant conclusion on the biologic behavior of these tumors is not possible because of the small number of cases described. However, factors that affect survival outcome in conventional ACC as reported by Gomez et al. [6] include perineural invasion, high-grade tumors in addition to positive surgical margins [25].

 Thompson et al. [17] reported median survival for this type of carcinoma 2.2 years, despite vigorous therapeutic intervention.

## 4. Conclusion

In this case report, we present a middle-aged woman with an unusual high-grade transformed variant of acinic cell carcinoma, which, as depicted in this case, tends to be more invasive and highly aggressive in its clinical course and outcome. Even though there are no standard guidelines on how to treat these salivary gland cancers, but what can be considered because of its nature, this tumor warrants the need for intense treatment regimens such as chemoradiation along with lymph node dissection in addition to the initial adequate surgical excision and frequent check-ups to identify recurrence and metastases.
